# Uterine rupture risk during trial of labor after one cesarean in a population-based cohort study of induction method and labor management

**DOI:** 10.1038/s41598-026-48444-z

**Published:** 2026-04-15

**Authors:** Christina Roeck Hansen, Ängla Mantel, Ingela Hulthén-Varli, Kari Johansson, Charlotte Lindblad Wollmann

**Affiliations:** 1 Clinical Epidemiology Division, Department of Medicine Solna, Karolinska Institutet, Stockholm, Sweden; 2 Division of Obstetrics and Gynecology, Department of Women’s and Children’s Health, Karolinska University Hospital, Stockholm, Sweden; 3 Department of Women’s and Children’s Health, Karolinska Institutet, Stockholm, Sweden

**Keywords:** Cohort study, Uterine rupture, Trial of labor after cesarean, Induction of labor, Maternal morbidities, Diseases, Health care, Medical research, Risk factors

## Abstract

A prior cesarean section is the primary risk factor for uterine rupture in a trial of labor after cesarean, a rare event associated with severe maternal and neonatal morbidity and mortality. This population-based cohort study aimed to determine the risk of uterine rupture in women undergoing trial of labor after one previous cesarean section in the Stockholm-Gotland Region and to examine associations between induction methods, labor management, and rupture risk. The cohort included 11,947 women with a cephalic-presenting, singleton infant at ≥ 37 + 0/6 weeks of gestation. Incidence of uterine rupture was calculated by labor onset and management strategies. Multivariable logistic regression assessed associations between labor characteristics, uterine rupture, and adverse maternal and perinatal outcomes. Overall, 216 (1.8%) women experienced uterine rupture. Induction of labor was associated with higher odds of rupture than spontaneous onset (aOR 1.63; 95% CI 1.20–2.22). Prostaglandin induction showed 2.6-fold increased odds (aOR 2.58; 95% CI 1.77–3.74), while balloon catheter showed no association (aOR 0.99; 95% CI 0.61–1.61). Prostaglandin use was thus linked to increased risk of uterine rupture, whereas balloon catheter induction was not. When induction is necessary, mechanical methods may be safer, though vigilant monitoring remains crucial.

**Supplementary Information:**

The online version contains supplementary material available at 10.1038/s41598-026-48444-z.

## Introduction

The mode of a woman’s first delivery has significant implications for her future reproductive health, influencing subsequent delivery options and serving as a key determinant of obstetric risk. Notably, a prior cesarean section (CS) is the primary risk factor for uterine rupture during a subsequent trial of labor^1^. Although rare, uterine rupture is a potentially catastrophic obstetric event associated with severe maternal and neonatal morbidity and mortality^2,3^.

Current clinical guidelines from the Royal College of Obstetricians and Gynaecologists (RCOG) and the American College of Obstetricians and Gynecologists (ACOG), among others, recommend that women with one previous CS may be offered a trial of labor after cesarean (TOLAC)^4–6^. Both spontaneous labor onset and labor induction are considered acceptable approaches; however, both have been associated with an increased risk of uterine rupture^7–10^.

With cesarean rates rising globally—now accounting for 21% of all births worldwide^11^—and induction and augmentation of labor becoming increasingly common^4^, the need for robust, evidence-based strategies to manage TOLAC is more urgent than ever. In Sweden, for example, 30% of deliveries are induced, and more than half of all women in labor receive some form of augmentation according to annual reports of national statistics in 2024^4^.

Studying uterine rupture is inherently challenging due to its low incidence. Randomized controlled studies are scarce, and existing evidence is largely derived from retrospective studies with heterogeneous study designs and inclusion criteria, often yielding inconsistent findings^1,12^. This variability has contributed to ongoing debate regarding optimal risk stratification and management strategies for women undergoing TOLAC^7,13–16^. The objective of this study was to investigate the association between labor management strategies—including induction methods—and the risk of uterine rupture, as well as to examine related perinatal outcomes, using a large contemporary population-based cohort with prospectively collected clinical information.

## Methods

### Setting and data sources

In Sweden, maternal health services are publicly funded and provided free of charge, resulting in near-universal coverage with 98.9%^4^ of pregnant women participating. The Stockholm-Gotland Perinatal Cohort (SGPC) is a prospectively collected, population-based cohort including the eight delivery centers across the Stockholm and Gotland regions in Sweden—representing ~ 25% of Swedish births^17^. It integrates data from electronic medical records, including antenatal visits and admissions, labor and delivery, neonatal care, and postpartum follow-up. Personal identification numbers have been replaced by pseudo-anonymous serial numbers from the Swedish National Board of Health and Welfare. All data management and analysis were performed on de-identified data.

The study was approved by the Swedish Ethical Review Authority, before 2019 named the Regional Ethical Review Board in Stockholm (2009/275-31, 2012/365-32, 2013/792-32, 2014/177-32, 2014/962-32, 2019-02818, 2020-01162, 2021-01229). According to these approvals, the requirement for informed consent was waived.

All methods and analyses were performed in accordance with relevant guidelines and regulations.

Diagnoses and procedures in SGPC are registered according to the International Classification of Diseases version 10 (*ICD-10*).

### Study population

We identified 335,153 births from 22 + 0/6 gestational weeks onward from the SGPC between 2008 and June 2020. From this group, we included women with a first (primary) CS, followed by a subsequent singleton delivery during the same time period. The second delivery was restricted to live-born, cephalic-presenting infants at ≥ 37 + 0/6 weeks of gestation, yielding an initial cohort of 21,214 women.

**Fig. 1:**
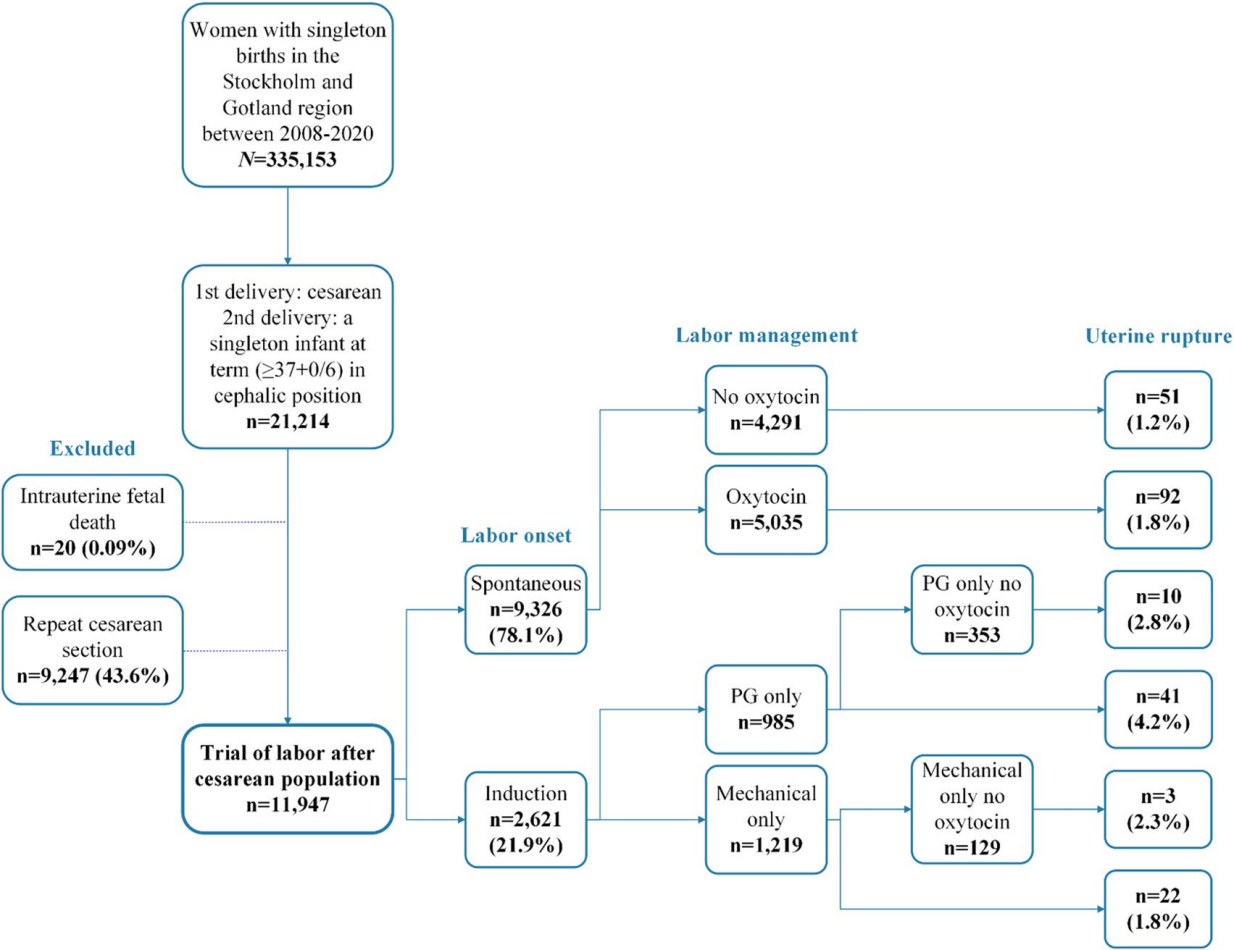
Flowchart of the study population and then stratified by onset of delivery: spontaneous onset, augmentation, and method of induction. PG, prostaglandins.

We excluded women with a planned (elective) CS, as registered in the SGPC, in the second pregnancy (*n* = 9247), as well as those who experienced intrauterine fetal death (*n* = 20), resulting in a final study population of 11,947 women who underwent TOLAC (see Fig. 1).

### Exposures and covariates

The primary exposure was labor induction versus spontaneous onset. Induction methods were further categorized as medical, using prostaglandins (PG); including Misoprostol (a synthetic PGE1 analogue) and Dinoprostone (a PGE2 analogue), or mechanical induction using a transcervical balloon catheter. These methods were identified by ICD-10 [O610, O610A, O610B, and O610X for medical induction; O611, O611A, O611B, or O611X for mechanical induction] combined with registry checkboxes specifying the induction method used. Oxytocin use was also recorded and compared between the groups (Fig. 1).

Baseline descriptive data, including maternal risk factors and characteristics (age, height, body mass index [BMI], smoking, snuff, cohabiting status, in-vitro-fertilization [IVF]), pregnancy complications and comorbidities (diabetes and hypertensive disorder noted by checkbox by the maternal health care provider or by ICD-10 [O24.0, O24.1, O24.4, O10, O13.9, O14 or O15]) were collected. Delivery characteristics (mode of delivery, induction methods, epidural anesthesia, as well as gestational age and birth weight) were also collected. Data on Bishop score, as recorded at the start of induction, were obtained for the induction groups.

### Outcomes

The primary outcome was uterine rupture, identified as registered diagnosis in the SGPC (ICD-10; O71.0 or O71.1). The attending clinician established the diagnosis during emergency CS or postpartum laparotomy performed in response to suspected complications. To reduce the risk of outcome misclassification, particularly the inclusion of uterine dehiscence cases identified through registry coding, we performed a supplementary sensitivity analysis using a stricter, clinically oriented definition of uterine rupture. Severe uterine rupture was defined as a registered diagnosis of uterine rupture in combination with the composite outcome of neonatal asphyxia (metabolic acidosis or Apgar score < 4 at 5 min).

Secondary maternal outcomes included postpartum hemorrhage, defined as an estimated blood loss > 1000 ml and hysterectomy, identified through registered diagnostic or procedural codes (ICD-10; O82.2, LCD00, LCD30, or MCA33).

Neonatal secondary outcomes included Apgar score < 4 at 5 min, umbilical cord artery pH < 7.0, metabolic acidosis defined as pH < 7.0 or pH < 7.10 in combination with base excess <-12 in umbilical cord artery blood, and a composite outcome of asphyxia defined as either metabolic acidosis (as above) or Apgar < 4 at 5 min. Additional neonatal outcomes were hypoxic ischemic encephalopathy^15^(ICD-10; P91.6, P91.6 A, P91.6B, P91.6 C or P91.6X), and neonatal seizure (ICD-10; P909, P909A, P909B or P909C).

### Statistical analysis

Maternal, neonatal, and delivery characteristics were tabulated and compared between women with versus without uterine rupture (primary outcome). Categorical variables were analyzed using the chi-square test, and continuous variables were analyzed using the two-tailed Student’s t test when normally distributed; otherwise, the Kruskal-Wallis test was performed. A p-value < 0.05 was considered statistically significant. The proportion of missing data was low (< 5%). Descriptive statistics were based on available data.

Due to the rarity of the primary outcome, crude and multivariable logistic regression analyses were performed using complete-case data to calculate odds ratios (ORs) with 95% confidence intervals (CIs) to estimate the associations between labor characteristics and uterine rupture, as well as neonatal outcomes. Potential confounders were selected based on prior knowledge and clinical experience. They included maternal height, maternal age, BMI, gestational age, year of delivery, and delivery clinic when analyzing for delivery onset characteristics and labor management. Further adjustment for Bishop score was added for comparison between the induction groups. Maternal age, BMI, gestational age, year of delivery, and maternal morbidities (hypertension disorders and diabetes) were considered when analyzing for neonatal outcomes.

Statistical analyses were conducted using SAS^®^ software version 9.4 (SAS Institute Inc., Cary, NC, USA).

## Results

### Incidence and labor characteristics

Among the 11,947 women undergoing TOLAC, the overall incidence of uterine rupture was 1.8% (*n* = 216).

Baseline maternal and labor characteristics are presented in Table 1. Women with uterine rupture had significantly higher BMI, were more likely to receive epidural anesthesia, as well as undergo repeat cesarean section and induction of labor. There was specifically a higher rate of induction of labor using prostaglandins in the uterine rupture group. Among women undergoing induction of labor, Bishop score distribution differed according to rupture status. The women with uterine rupture also had higher rates of postpartum hemorrhage and hysterectomy. Additionally, these pregnancies tended to have longer gestational duration and higher neonatal birthweights. In the supplementary sensitivity analysis using the stricter outcome definition (severe uterine rupture), some baseline characteristics differed between the groups (Supplementary Table S1). There were 52 cases of severe uterine rupture (0.4%). Women with severe uterine rupture were shorter than those without rupture. Otherwise, no statistically significant difference was observed between the groups regarding induction of labor, Bishop score distribution among those with prostaglandin induction, hysterectomy, gestational length, or birth weight, in contrast to the analysis using the broader definition of uterine rupture.

**Table 1:** Maternal and perinatal characteristics by uterine rupture in women with trial of labor after a first cesarean delivery, Stockholm-Gotland, Sweden 2008–2020 (*N* = 11,947).

	Uterine rupture					
	**No** *n* = 11,731 (98.2%)		**Yes** *n* = 216 (1.8%)		*p*-value	Missing data (%)
	n	%	n	%		
**Maternal characteristics**						
Age mean years (± SD)	32.7	(4.5)	32.7	(4.5)	0.79	7 (0.1)
Age years					0.84	
≤ 19	16	0.1	0	0.0		
20–29	2811	24.0	51	23.6		
30–34	4760	40.6	86	39.8		
35–39	3467	29.6	63	29.2		
≥ 40	670	5.7	16	7.4		
Height mean cm (± SD)	164.9	(6.6)	164.1	(6.0)	0.10	134 (1.1)
Height cm					0.16	
≤ 154	667	5.8	14	6.6		
155–164	4838	41.7	93	44.1		
165–174	5249	45.3	97	46.0		
≥ 175	847	7.3	7	3.3		
BMI mean kg/m^2^ (± SD)	24.8	(4.5)	25.5	(4.9)	0.02	455 (3.8)
BMI kg/m^2^					0.03	
≤ 19.9	980	8.9	17	8.5		
20.0–24.9	5654	51.1	81	40.7		
25.0–29.9	3027	27.4	70	35.2		
30.0–34.9	1011	9.1	20	10.1		
≥ 35.0	391	3.5	11	5.5		
Hypertensive disease	589	5.0	8	3.7	0.38	–
Diabetes	329	2.8	8	3.7	0.43	–
IVF	515	4.4	13	6.0	0.25	–
Smoking	309	2.7	6	2.8	0.91	178 (1.5)
Snuff	81	0.7	< 5	0.9	0.68	–
Cohabiting with partner	11,097	96.1	208	97.7	0.24	184 (1.5)
**Delivery characteristics**						
Mode of delivery					< 0.001	–
* Cesarean section*	3596	30.7	191	88.4		
*Vaginal delivery*	8135	69.4	25	11.6		
*Instrumental vaginal delivery*	1458	12.4	16	7.4		
Induction of labor	2548	21.7	73	33.8	< 0.001	–
*Mechanical*	1197	10.2	22	10.2	0.99	–
*Bishop score*					0.03	73 (6.0)
*0–3*	331	29.2	4	19.1		–
*4–5*	441	39.2	12	57.1		
*>5*	353	31.4	5	23.8		
*Prostaglandin*	944	8.1	41	19.0	< 0.001	
*Bishop score*					0.02	49 (5.0)
*0–3*	505	56.3	13	33.3		
*4–5*	234	26.1	15	38.5		
*>5*	158	17.6	11	28.2		
Hemorrhage > 1000 ml	960	8.2	60	28.3	< 0.001	52 (0.4)
Hysterectomy	0	0.0	< 5	0.5	< 0.001	–
Epidural	7255	61.8	176	81.5	< 0.001	–
Gestational length mean days (± SD)	281	(9)	284	(8)	< 0.001	–
Birth weight mean g (+/-SD)	3608	(480)	3697	(481)	0.007	18 (0.2)
Macrosomia (> 4500 g)	390	3.3	10	4.7	0.29	–

For outcome < 5 exact numbers are not given.

SD, standard deviation; BMI, body mass index; IVF, in vitro fertilization.

### Labor onset, induction methods, and uterine rupture

An overview of labor onset and management, stratified by spontaneous vs. induced onset and their respective management pathways is presented in Fig. 1.

In total, 9326 (78.1%) women had a spontaneous onset of labor, of whom 143 (1.5%) experienced uterine rupture. Within this group, 4291 (46%) labored without oxytocin augmentation, while 5035 (54%) received oxytocin augmentation. The incidence of uterine rupture was 1.2% in the non-augmented group and 1.8% in the augmented group, corresponding to 1.5-fold increased odds of uterine rupture. However, this association did not remain statistically significant after adjustment for potential confounders.

In contrast, 2621 (21.9%) women underwent induction of labor, among whom 73 (2.8%) experienced uterine rupture. Compared with spontaneous onset (with or without oxytocin), induction of labor was associated with 1.6-fold increased odds of uterine rupture (aOR 1.63; 95% CI 1.20–2.22).

When evaluating induction methods separately, prostaglandin use (with or without oxytocin) was associated with the highest odds of uterine rupture. Among 985 women induced with prostaglandins, 41 (4.2%) experienced uterine rupture. Compared with spontaneous labor onset, prostaglandin induction was associated with 2.6-fold higher adjusted odds of uterine rupture (aOR 2.58; 95% CI 1.77–3.74). Among 1219 women induced mechanically, 22 (1.8%) experienced uterine rupture, which did not differ significantly from spontaneous onset (aOR 0.99; 95% CI 0.61–1.61). When comparing prostaglandins and mechanical induction separately to spontaneous onset without the usage of oxytocin, both associations were non-significant (aOR 1.92; 95% CI 0.89–4.12 and aOR 1.09; 95% CI 0.26–4.62 respectively). However, when directly compared, prostaglandin induction was associated with 3-fold higher adjusted odds of uterine rupture than mechanical induction (aOR 3.07; 95% CI 1.73–5.48) (Table 2). In the supplementary sensitivity analysis using severe uterine rupture as the outcome, the direction of the associations was consistent with the main analysis (Supplementary Table S2). Compared with spontaneous labor, prostaglandin induction remained associated with a significantly increased odds of severe uterine rupture (aOR 2.31; 95% CI 1.07–4.99). Similarly, prostaglandin induction was associated with 3.5-fold higher odds of severe uterine rupture compared with mechanical induction (aOR 3.48; 95% CI 1.01–12.02), whereas the remaining comparisons did not reach statistical significance.

**Table 2:** Labor onset and management characteristics, and their association with uterine rupture in a Swedish cohort of women undergoing trial of labor after cesarean (*N* = 11,947).

Labor characteristics	No. of patients	Uterine rupture	Odds ratio crude		Odds ratio adjusted^b^		*p*-value
		No. (%)	cOR	95% CI	aOR	95% CI	
Spontaneous (Ref)^a^	9326	143 (1.5)	–	–	–	–	–
IOL	2621	73 (2.8)	1.84	1.38–2.45	1.63	1.20–2.22	0.002
IOL with PG only	985	41 (4.2)	2.79	1.96–3.97	2.58	1.77–3.74	< 0.001
Mechanical IOL only	1219	22 (1.8)	1.18	0.75–1.86	0.99	0.61–1.61	0.96
Spontaneous, no oxytocin (Ref)^c^	4291	51(1.2)	–	–	–	–	–
Augmented	5035	92 (1.8)	1.55	1.10–2.18	1.35	0.94–1.93	0.12
IOL with PG only, no oxytocin	353	10 (2.8)	2.42	1.22–4.82	1.92	0.89–4.12	0.09
Mechanical IOL only, no oxytocin	129	< 5	1.98	0.61–6.43	1.09	0.26–4.62	0.91
Mechanical IOL only (Ref)^d^	1219	22 (1.8)	–	–	–	–	–
IOL with PG only	985	41 (4.2)	2.36	1.40–4.00	3.07	1.73–5.48	< 0.001

^a^Women with spontaneous onset of labor with or without the use of oxytocin served as reference group.

^b^Adjusted for maternal height, maternal age, body mass index (BMI), gestational age, year of delivery, and delivery clinic.

^c^Women with spontaneous onset of labor without the use of oxytocin served as reference group.

^d^Women undergoing induction of labor using mechanical methods only with or without the use of oxytocin served as reference group. The model is additionally adjusted for Bishop score.

IOL, induction of labor; PG, prostaglandin.

### Perinatal outcomes

All adverse neonatal outcomes assessed were significantly more frequent among infants born to mothers who experienced uterine rupture. As shown in Table 3, uterine rupture was strongly associated with increased odds of severe neonatal morbidity. An Apgar score < 4 at 5 min occurred in 5.1% of infants in the uterine rupture group, corresponding to a 20-fold increase in adjusted odds compared with infants born without rupture (aOR 20.25; 95% CI 9.92–41.35). Umbilical cord artery pH < 7.0 was observed in 19.3% (aOR 16.95; 95% CI 10.87–26.43), and metabolic acidosis in 27.7% (aOR 9.86; 95% CI 6.79–14.33). The composite outcome of asphyxia occurred in 24.1% (aOR 10.42; 95% CI 7.38–14.73). Neonatal seizures (3.2%) and HIE (4.6%) were likewise associated with uterine rupture (aOR 16.21; 95% CI 6.38–41.17 and aOR 15.12; 95% CI 6.74–33.89, respectively).

**Table 3:** Neonatal outcomes associated with uterine rupture in a Swedish cohort of women undergoing trial of labor after cesarean (*N* = 11,947).

Neonatal outcome	No. of patients	Uterine rupture	Odds ratio crude		Odds ratio adjusted^b^		*p*-value
		No. (%)	cOR	95% CI	aOR	95% CI	
No uterine rupture (Ref) ^a^	11,731	–	–	–	–	–	–
Apgar at 5 min < 4	42	11 (5.1)	20.23	10.03–40.81	20.25	9.92–41.35	< 0.001
Umbilical cord artery pH < 7.0	140	34 (19.3)	18.63	12.24–28.37	16.95	10.87–26.43	< 0.001
Metabolic acidosis^c^	350	47 (27.7)	10.03	7.03–14.31	9.86	6.79–14.33	< 0.001
Compound asphyxia^d^	382	52 (24.1)	10.96	7.87–15.25	10.42	7.38–14.73	< 0.001
Neonatal seizure	31	7 (3.2)	16.34	6.96–38.34	16.21	6.38–41.17	< 0.001
HIE	42	10 (4.6)	17.75	8.61–36.58	15.12	6.74–33.89	< 0.001

^a^Women without uterine rupture used as reference group.

^b^Adjusted for maternal age, body mass index (BMI), gestational age, year of delivery, maternal hypertensive disease, and diabetes.

^c^Metabolic acidosis: umbilical artery pH < 7.0 or pH < 7.10 and umbilical artery base.

excess<-12.

^d^Compound asphyxia: metabolic acidosis or Apgar at 5 min < 4.

HIE, hypoxic ischemic encephalopathy.

## Discussion

In this large contemporary population-based cohort of 11,947 women with one previous cesarean undergoing TOLAC, the incidence of uterine rupture was 1.8%. Induction of labor was associated with higher odds of uterine rupture compared with spontaneous labor onset, particularly when prostaglandins were used. Uterine rupture was further linked to substantial maternal and neonatal morbidity.

A major strength of this study is the use of a large, population-based cohort of nearly 12,000 women across eight delivery centers, representing one quarter of all births in Sweden during the study period. The near-universal coverage of antenatal care in Sweden, and the high proportion of hospital-based deliveries, further enhance the generalizability of our findings to other similar high-income settings. Given the rarity of uterine rupture and the ethical and logistical challenges of conducting randomized controlled trials in this context, large-scale observational data are critical for generating clinically relevant evidence. This study provides robust estimates of uterine rupture risk across different modes of labor onset and induction strategies, directly informing clinical practice in settings where TOLAC is widely recommended.

Limitations include missing details on the index cesarean delivery and prior uterine surgery, which may affect the risk of future uterine rupture^10,18,19^. Information on dose and route of prostaglandin administration was unavailable, precluding evaluation of their potential influence on uterine rupture risk. The use of oxytocin was not statistically significantly associated with uterine rupture in the present study, which differs from previous reports, including the meta-analysis by Zhang et al.^7^ suggesting an increased risk associated with oxytocin use during TOLAC. That meta-analysis also highlights the potential importance of dose-response relationships between oxytocin exposure and uterine rupture. Information on oxytocin dosage and duration was not available in the present dataset, which limits the interpretation of these findings. Finally, analyses evaluating oxytocin as a potential mediator may have provided additional insight but were beyond the scope of the current study. The diagnosis of uterine rupture relied on ICD-10 codes recorded by the attending physician, which may introduce misclassification, for example, where partial uterine ruptures or uterine dehiscence were included. However, the supplementary sensitivity analysis restricting the outcome to severe uterine rupture supports the robustness of our main findings. The association between prostaglandin induction and uterine rupture, compared to both spontaneous labor onset and mechanical induction, remained statistically significant, validating the main findings in this study. Finally, some neonatal outcomes may have been underreported if diagnosed after transfer to a neonatal unit, although uterine rupture is typically captured at delivery.

This study highlights the risk of substantial maternal and neonatal morbidity, including neonatal asphyxia, associated with uterine rupture. The incidence of uterine rupture in our study (1.8%) was higher than the incidences found in many previous studies, which typically range from 0.5 to 0.9% among women with a previous low-transverse uterine incision^5^. One potential explanation is that our population consisted exclusively of women with one previous cesarean and no prior vaginal deliveries, whereas many previous studies reporting a lower incidence of uterine rupture include women with prior vaginal deliveries. Previous vaginal delivery, including successful TOLAC, has consistently been shown to reduce the risk of uterine rupture^8^. A study by Zelop et al.^20^demonstrated an incidence of 1.1% for uterine rupture among women undergoing TOLAC with no prior vaginal delivery. Consequently, studies including multiparous women with prior vaginal deliveries may underestimate the risk for first-time TOLAC candidates, whereas our findings may be more representative of this higher-risk subgroup. The use of ICD-codes for the identification of uterine rupture also introduces the risk of including partial ruptures and dehiscence, as previously discussed, which may lead to an inflation of the overall incidence. However, the strong association observed in this study with neonatal morbidity, as well as the supplementary sensitivity analysis support the clinical relevance of the recorded diagnoses. In a Norwegian registry-based study, in which uterine rupture diagnoses were clinically verified, Al-Zirqi et al.^21^ reported that the temporal trend of uterine rupture appears to be rising, which may partly reflect changes in obstetric care during TOLAC, such as increasing rates of labor induction and augmentation use. The combination of the high-risk population in this study, broader classification of uterine rupture, and evolving clinical practice may therefore explain the higher incidence of uterine rupture found in this study.

The association between labor induction and the risk of uterine rupture has been examined in multiple studies, yet findings remain inconsistent. A systematic review and meta-analysis by Wingert et al.^22^ highlighted important limitations in the evidence base, noting that most studies suffer from small sample sizes and low event rates, limiting the precision of risk estimates and the ability to provide evidence-based counselling for considering TOLAC. Similarly, a review by Deshmukh et al.^23^ indicated an increased risk of uterine rupture associated with induction of labor, particularly when prostaglandins were used. Interpretation, however, was complicated by the frequent concomitant use of oxytocin, making it difficult to isolate the specific contribution of each agent. Many previous studies were also single-center and retrospective studies with heterogeneous induction protocols and exposure definitions. This study shares some of these limitations; however, by contrast, the large multicenter design of the present study and the ability to analyze prostaglandins and balloon catheter separately, strengthen the validity of our findings.

Another important factor influencing both induction strategy and uterine rupture risk is cervical status at the time of induction. A more favorable Bishop score has been associated with shorter labor duration, whereas prolonged labor duration increases the likelihood of uterine rupture^24,25^. Women with an unfavorable cervix often require prostaglandins for cervical ripening^26,27^. Prostaglandins, however, have been linked to an increased risk of uterine hyperstimulation^27,28^, which may in turn increase intrauterine pressure and mechanical stress on the uterine scar. Thus, the elevated risk observed with prostaglandins may therefore reflect a combination of drug effects and cervical factors. However, adjustment for Bishop score was included when analyzing the association between prostaglandin induction and uterine rupture compared with mechanical induction, indicating that the increased risk may not be explained by cervical favorability alone.

Future studies should aim to disentangle the effects of prostaglandin type and dosage, and to evaluate the mediating role of oxytocin in the pathway to uterine rupture.

## Conclusion

In this large population-based cohort of women with one previous CS, induction of labor was associated with higher odds of uterine rupture compared with spontaneous labor onset. The risk was particularly elevated with prostaglandin use, whereas mechanical induction with a balloon catheter was not associated with increased odds.

For women undergoing TOLAC, spontaneous labor onset remains the safest option. When induction is necessary, careful monitoring is essential, and mechanical methods may represent a safer alternative to minimize the risk of uterine rupture and its associated maternal and neonatal morbidity.

## Supplementary Information

Below is the link to the electronic supplementary material .

Supplementary Material 1

## Data Availability

The data that support the findings of this study are not publicly available due to ethical and legal restrictions, as they contain sensitive health information and were accessed under approval from regional ethical boards and data-holding authorities. De-identified data may be made available to qualified researchers after approval by the Swedish Ethical Review Authority and with permission from the relevant data holders. The statistical analysis code (SAS) is available from the corresponding author upon reasonable request.

## References

[1] Guise, J. M. et al. Systematic review of the incidence and consequences of uterine rupture in women with previous caesarean section. *BMJ (Clinical Res. ed.)*. **329**, 19–25. 10.1136/bmj.329.7456.19 (2004). 10.1136/bmj.329.7456.19 PMC443444 15231616

[2] Hofmeyr, G. J., Say, L. & Gulmezoglu, A. M. WHO systematic review of maternal mortality and morbidity: The prevalence of uterine rupture. *BJOG* **112**, 1221–1228. 10.1111/j.1471-0528.2005.00725.x (2005). 16101600 10.1111/j.1471-0528.2005.00725.x

[3] Guiliano, M. et al. Signs, symptoms and complications of complete and partial uterine ruptures during pregnancy and delivery. *Eur. J. Obstet. Gynecol. Reprod. Biol.* **179**, 130–134. 10.1016/j.ejogrb.2014.05.004 (2014). 24965993 10.1016/j.ejogrb.2014.05.004

[4] Graviditetsregistret Årsrapport (Graviditetsregistret, Sverige, 2025). (2024).

[5] Obstetricians, A. C. o. & Gynecologists. Vaginal birth after cesarean delivery. *Committee Opinion* **64** (1988).

[6] Royal College of Obstetricians and Gynaecologists. *Birth After Previous Caesarean Birth. Green-top Guideline No. 45* (Royal College of Obstetricians and Gynaecologists, 2015).

[7] Zhang, H., Liu, H., Luo, S. & Gu, W. Oxytocin use in trial of labor after cesarean and its relationship with risk of uterine rupture in women with one previous cesarean section: A meta-analysis of observational studies. *BMC Pregnancy Childbirth* **21**, 11. 10.1186/s12884-020-03440-7 (2021). 33407241 10.1186/s12884-020-03440-7 PMC7786988

[8] Grobman, W. A. et al. Prediction of uterine rupture associated with attempted vaginal birth after cesarean delivery. *Am. J. Obstet. Gynecol.* **199**, 30e31–30e35. 10.1016/j.ajog.2008.03.039 (2008). 10.1016/j.ajog.2008.03.039 PMC2532505 18439555

[9] Landon, M. B. et al. Risk of uterine rupture with a trial of labor in women with multiple and single prior cesarean delivery. *Obstet. Gynecol.* **108**, 12–20. 10.1097/01.AOG.0000224694.32531.f3 (2006). 16816050 10.1097/01.AOG.0000224694.32531.f3

[10] Al-Zirqi, I., Daltveit, A. K., Forsen, L., Stray-Pedersen, B. & Vangen, S. Risk factors for complete uterine rupture. *Am. J. Obstet. Gynecol.* **216**, 165 e161–165 e168. 10.1016/j.ajog.2016.10.017 (2017). 10.1016/j.ajog.2016.10.017 27780708

[11] Betran, A. P., Ye, J., Moller, A. B., Souza, J. P. & Zhang, J. Trends and projections of caesarean section rates: Global and regional estimates. *BMJ Glob. Health*. **6**. 10.1136/bmjgh-2021-005671 (2021). 10.1136/bmjgh-2021-005671 PMC8208001 34130991

[12] West, H. M., Jozwiak, M. & Dodd, J. M. Methods of term labour induction for women with a previous caesarean section. *Cochrane Datab. Syst. Rev*. 10.1002/14651858.CD009792.pub3 (2017). 10.1002/14651858.CD009792.pub3 PMC6481365 28599068

[13] Harper, L. M. et al. Association of induction of labor and uterine rupture in women attempting vaginal birth after cesarean: A survival analysis. *Am. J. Obstet. Gynecol.* **206**, 51e51–51e55. 10.1016/j.ajog.2011.09.022 (2012). 10.1016/j.ajog.2011.09.022 PMC3246100 22000899

[14] Wallstrom, T. et al. Induction of labor after one previous Cesarean section in women with an unfavorable cervix: A retrospective cohort study. *PLoS One*. **13**, e0200024. 10.1371/journal.pone.0200024 (2018). 29965989 10.1371/journal.pone.0200024 PMC6028115

[15] Bujold, E., Blackwell, S. C. & Gauthier, R. J. Cervical ripening with transcervical foley catheter and the risk of uterine rupture. *Obstet. Gynecol.* **103**, 18–23. 10.1097/01.AOG.0000109148.23082.C1 (2004). 14704239 10.1097/01.AOG.0000109148.23082.C1

[16] Lydon-Rochelle, M., Holt, V. L., Easterling, T. R. & Martin, D. P. Risk of uterine rupture during labor among women with a prior cesarean delivery. *N. Engl. J. Med.* **345**, 3–8. 10.1056/NEJM200107053450101 (2001). 11439945 10.1056/NEJM200107053450101

[17] Johansson, K. et al. The Stockholm-Gotland perinatal cohort—A population-based cohort including longitudinal data throughout pregnancy and the postpartum period. *Paediatr. Perinat. Epidemiol.* **37**, 276–286. 10.1111/ppe.12945 (2023). 36560891 10.1111/ppe.12945

[18] Stamilio, D. M. et al. Short interpregnancy interval: Risk of uterine rupture and complications of vaginal birth after cesarean delivery. *Obstet. Gynecol.* **110**, 1075–1082. 10.1097/01.Aog.0000286759.49895.46 (2007). 17978122 10.1097/01.AOG.0000286759.49895.46

[19] Claeys, J., Hellendoorn, I., Hamerlynck, T., Bosteels, J. & Weyers, S. The risk of uterine rupture after myomectomy: A systematic review of the literature and meta-analysis. *Gynecol. Surg.* **11**, 197–206. 10.1007/s10397-014-0842-8 (2014).

[20] Zelop, C. M., Shipp, T. D., Repke, J. T., Cohen, A. & Lieberman, E. Effect of previous vaginal delivery on the risk of uterine rupture during a subsequent trial of labor. *Am. J. Obstet. Gynecol.* **183**, 1184–1186. 10.1067/mob.2000.109048 (2000). 11084564 10.1067/mob.2000.109048

[21] Al-Zirqi, I., Stray-Pedersen, B., Forsen, L., Daltveit, A. K. & Vangen, S. Uterine rupture: Trends over 40 years. *BJOG* **123**, 780–787. 10.1111/1471-0528.13394 (2016). 25846698 10.1111/1471-0528.13394

[22] Wingert, A. et al. Clinical interventions that influence vaginal birth after cesarean delivery rates: Systematic review & meta-analysis. *BMC Pregnancy Childbirth*. **19**. 10.1186/s12884-019-2689-5 (2019). 10.1186/s12884-019-2689-5 PMC6937863 31888540

[23] Deshmukh, U., Denoble, A. E. & Son, M. Trial of labor after cesarean, vaginal birth after cesarean, and the risk of uterine rupture: An expert review. *Am. J. Obstet. Gynecol.* **230**, S783–s803. 10.1016/j.ajog.2022.10.030 (2024). 38462257 10.1016/j.ajog.2022.10.030

[24] Teixeira, C., Lunet, N., Rodrigues, T. & Barros, H. The Bishop Score as a determinant of labour induction success: A systematic review and meta-analysis. *Arch. Gynecol. Obstet.* **286**, 739–753. 10.1007/s00404-012-2341-3 (2012). 22546948 10.1007/s00404-012-2341-3

[25] Hesselman, S. et al. Time matters-a Swedish cohort study of labor duration and risk of uterine rupture. *Acta Obstet. Gynecol. Scand.* **100**, 1902–1909. 10.1111/aogs.14211 (2021). 34114644 10.1111/aogs.14211

[26] Mackenzie, I. Z. Induction of labour at the start of the new millennium. *Reprod. (Cambridge England)*. **131**, 989–998. 10.1530/rep.1.00709 (2006). 10.1530/rep.1.00709 16735538

[27] Rayburn, W. F. Preinduction cervical ripening: Basis and methods of current practice. *Obstet. Gynecol. Surv.* **57**, 683–692. 10.1097/00006254-200210000-00022 (2002). 12368596 10.1097/00006254-200210000-00022

[28] Justus Hofmeyr, G. Induction of labour with an unfavourable cervix. *Best Pract. Res. Clin. Obstet. Gynecol.* **17**, 777–794. 10.1016/S1521-6934(03)00037-3 (2003). 10.1016/s1521-6934(03)00037-3 12972014

