# Supplementary material for: Uterine rupture risk during trial of labor after one cesarean in a population-based cohort study of induction method and labor management
Source: Sci Rep. 2026 Apr 15;16:12473. doi: 10.1038/s41598-026-48444-z (PMC13087215; doi:10.1038/s41598-026-48444-z)
Supplement: Supplementary file 1 — Supplementary Material 1 [file 41598_2026_48444_MOESM1_ESM.docx]

**Supplementary Table S1.** Maternal and perinatal characteristics by severe uterine rupture in women with trial of labor after a first cesarean delivery, Stockholm-Gotland, Sweden 2008-2020 (N=11,947)

|  | **Severe uterine rupture*** | | | |  |  |
| --- | --- | --- | --- | --- | --- | --- |
|  | **No** n=11,895 (99.6%) | | **Yes** n=52 (0.4%) | |  |  |
|  | n | % | n | % | p-value | Missing data (%) |
| **Maternal characteristics** |  |  |  |  |  |  |
| Age mean years (± SD) | 32.7 | (4.5) | 31.9 | (4.6) | 0.2 | 7 (0.1) |
| Age years |  |  |  |  | 0.54 |  |
| ≤19 | 16 | 0.1 | 0 | 0.0 |  |  |
| 20 – 29 | 2846 | 23.9 | 16 | 30.8 |  |  |
| 30 – 34 | 4823 | 40.6 | 23 | 44.2 |  |  |
| 35 – 39 | 3520 | 29.6 | 10 | 19.2 |  |  |
| ≥40 | 683 | 5.8 | <5 | 5.8 |  |  |
| Height mean cm (± SD) | 164.9 | (6.6) | 162.3 | (6.6) | 0.005 | 134 (1.1) |
| Height cm |  |  |  |  | 0.06 |  |
| ≤154 | 675 | 5.7 | 6 | 12.0 |  |  |
| 155 – 164 | 4908 | 41.7 | 23 | 46.0 |  |  |
| 165 – 174 | 5325 | 45.3 | 21 | 42.0 |  |  |
| ≥175 | 854 | 7.3 | 0 | 0 |  |  |
| BMI mean kg/m^2^ (± SD) | 24.8 | (4.5) | 26.2 | (5.2) | 0.03 | 455 (3.8) |
| BMI kg/m^2^ |  |  |  |  | 0.13 |  |
| ≤19.9 | 994 | 8.9 | <5 | 6.7 |  |  |
| 20.0 – 24.9 | 5718 | 51.0 | 17 | 37.8 |  |  |
| 25.0 – 29.9 | 3080 | 27.5 | 17 | 37.8 |  |  |
| 30.0 – 34.9 | 1027 | 9.2 | <5 | 8.9 |  |  |
| ≥35.0 | 398 | 3.6 | <5 | 8.9 |  |  |
| Hypertensive disease | 596 | 5.0 | <5 | 1.9 | 0.31 | - |
| Diabetes | 335 | 2.8 | <5 | 3.9 | 0.65 | - |
| IVF | 524 | 4.4 | <5 | 7.7 | 0.25 | - |
| Smoking | 314 | 2.7 | <5 | 1.9 | 0.74 | 178 (1.5) |
| Snuff | 83 | 0.7 | 0 | 0.0 | 0.55 | - |
| Cohabiting with partner | 11256 | 96.1 | 49 | 98.0 | 0.49 | 184 (1.5) |
| **Delivery characteristics** |  |  |  |  |  |  |
| Mode of delivery |  |  |  |  | <0.001 | - |
| *Cesarean section* | 3740 | 31.4 | 47 | 90.4 |  |  |
| *Vaginal delivery* | 8155 | 68.6 | 5 | 9.6 |  |  |
| *Instrumental vaginal delivery* | 1470 | 12.4 | <5 | 7.7 |  |  |
| Induction of labor | 2605 | 21.9 | 16 | 30.8 | 0.12 | - |
| *Mechanical* | 1214 | 10.2 | 5 | 9.6 | 0.89 | - |
| *Bishop score* |  |  |  |  | 0.02 | 73 (6.0) |
| *0-3* | 335 | 29.4 | 0 | 0 |  |  |
| *4-5* | 448 | 39.3 | 5 | 100.0 |  |  |
| *>5* | 358 | 31.4 | 0 | 0 |  |  |

| *Prostaglandin* | 975 | 8.2 | 10 | 19.2 | 0.004 | - |
| --- | --- | --- | --- | --- | --- | --- |
| *Bishop score* |  |  |  |  | 0.9 | 49 (5.0) |
| *0-3* | 513 | 55.4 | 5 | 50.0 |  |  |
| *4-5* | 246 | 26.6 | <5 | 30.0 |  |  |
| *>5* | 167 | 18.0 | <5 | 20.0 |  |  |
| Hemorrhage >1000 ml | 1003 | 8.5 | 17 | 33.3 | <0.001 | 52 (0.4) |
| Hysterectomy | <5 | 0.01 | 0 | 0.0 | 0.95 | - |
| Epidural | 7385 | 62.1 | 46 | 88.5 | <0.001 | - |
| Gestational length mean days (± SD) | 281 | (9) | 283 | (9) | 0.29 | - |
| Birth weight mean g (+/-SD) | 3609 | (480) | 3652 | (502) | 0.52 | 18 (0.2) |
| Macrosomia (>4500g) | 399 | 3.4 | <5 | 2.0 | 0.58 |  |

**uterine rupture combined with the composite variable of neonatal asphyxia, defined as metabolic acidosis or Apgar at 5 min <4.*

*Note: For outcome <5, exact numbers are not given.*

*Abbreviations: SD, standard deviation; BMI, body mass index; IVF, in vitro fertilization*

**Supplementary Table S2.** Labor onset and management characteristics, and their association with severe uterine rupture in a Swedish cohort of women undergoing trial of labor after cesarean (N=11,947)

|  | **No. of patients** | **Severe uterine rupture*** | **Odds ratio**  **crude** | | **Odds ratio adjusted^b^** | | **p-value** |
| --- | --- | --- | --- | --- | --- | --- | --- |
| **Labor characteristics** |  | No. (%) | cOR | 95% CI | aOR | 95% CI |  |
| Spontaneous (Ref)^a^ | 9326 | 36 (0.4) | - | - | - | - | - |
| IOL | 2621 | 16 (0.6) | 1.59 | 0.88-2.86 | 1.28 | 0.67-2.45 | 0.46 |
| IOL with PG only | 985 | 10 (1.0) | 2.65 | 1.31-5.35 | 2.31 | 1.07-4.99 | 0.03 |
| Mechanical IOL only | 1219 | 5 (0.4) | 1.06 | 0.42-2.71 | 0.69 | 0.24-2.00 | 0.49 |
| Spontaneous, no oxytocin (Ref)^c^ | 4291 | 11(0.3) | - | - | - | - | - |
| Augmented | 5035 | 25 (0.5) | 1.94 | 0.95-3.95 | 1.72 | 0.81-3.65 | 0.16 |
| IOL with PG only, no oxytocin | 353 | 2 (0.6) | 2.22 | 0.49-10.04 | 0.70 | 0.08-6.14 | 0.75 |
| Mechanical IOL only, no oxytocin | 129 | 0 | - | - | - | - | - |
| Mechanical IOL only (Ref)^d^ | 1219 | 5 (0.4) | - | - | - | - | - |
| IOL with PG only | 985 | 10 (1.0) | 2.49 | 0.85-7.31 | 3.48 | 1.01-12.02 | 0.048 |

**uterine rupture combined with the composite variable of neonatal asphyxia, defined as metabolic acidosis or Apgar at 5 min <4.*

*^a^ women with spontaneous onset of labor with or without the use of oxytocin served as reference group.*

*^b^ adjusted for maternal height, maternal age, body mass index (BMI), gestational age, year of delivery, and delivery clinic.*

*^c^ women with spontaneous onset of labor without the use of oxytocin served as reference group.*

*^d^ women undergoing induction of labor using mechanical methods only, with or without the use of oxytocin, served as reference group. The model is additionally adjusted for Bishop score.*

*Abbreviations: IOL, induction of labor; PG, prostaglandin.*
